# Galectin-3 Predicts Long-Term Risk of Cerebral Disability and Mortality in Out-of-Hospital Cardiac Arrest Survivors

**DOI:** 10.3390/jpm14090994

**Published:** 2024-09-19

**Authors:** Amr Abdelradi, Wasim Mosleh, Sharma Kattel, Zaid Al-Jebaje, Arezou Tajlil, Saraswati Pokharel, Umesh C. Sharma

**Affiliations:** 1 Division of Cardiology, Department of Medicine, Jacob’s School of Medicine and Biomedical Sciences, University at Buffalo, Buffalo, NY 14068, USA; abdelrad@ualberta.ca (A.A.);; 2 Division of Cardiovascular Medicine, Department of Medicine, University of Minnesota, Minneapolis, MN 55455, USA; 3 Division of Cardiovascular Medicine, Department of Medicine, Henry Ford Health System, Detroit, MI 48202, USA; 4 Division of Thoracic Pathology and Oncology, Department of Pathology, Roswell Park Comprehensive Cancer Center, Buffalo, NY 14203, USA

**Keywords:** cardiac arrest, brain natriuretic peptide, galectin-3, mortality, neurological outcomes

## Abstract

Background: Out-of-hospital cardiac arrest (OHCA) is associated with high mortality and cerebral disability in survivors. Current models of risk prediction and survival are mainly based on resuscitation duration. We examined the prognostic value of circulating biomarkers in predicting mortality and severe cerebral disability for OHCA survivors, alongside traditional clinical risk indicators. Methods: Biomarkers including BNP, troponin I, and galectin-3 were measured at hospital admission in resuscitated OHCA patients. Prognostic significance for mortality and cerebral disability involving circulating biomarkers, resuscitation duration, demographics, and laboratory and clinical characteristics was examined via univariate and multivariate Cox proportional hazards regression models. The incremental prognostic value of the index covariates was examined through model diagnostics, focusing on the Akaike information criterion (AIC) and Harrell’s concordance statistic (c-statistic). Results: In a combinatorial analysis of 144 OHCA survivors (median follow-up 5.7 years (IQR 2.9–6.6)), BNP, galectin-3, arterial pH, and resuscitation time were significant predictors of all-cause death and severe cerebral disability, whereas troponin I levels were not. Multivariate regression, adjusting for BNP, arterial pH, and resuscitation time, identified galectin-3 as an independent predictor of long-term mortality. Multiple linear regression models also confirmed galectin-3 as the strongest predictor of cerebral disability. The incorporation of galectin-3 into models for predicting mortality and cerebral disability enhanced fit and discrimination, demonstrating the incremental value of galectin-3 beyond traditional risk predictors. Conclusions: Galectin-3 is a significant, independent long-term risk predictor of cerebral disability and mortality in OHCA survivors. Incorporating galectin-3 into current risk stratification models may enhance early prognostication and guide targeted clinical interventions.

## 1. Introduction

Out-of-hospital cardiac arrest (OHCA) manifests as sudden circulatory failure within an hour of symptom onset in individuals who otherwise appear healthy [1]. OHCA presents a significant challenge to public health wherein over 300,000 cases of OHCA with poor survival rates of 10–12% are reported annually in the United States [2]. Furthermore, among survivors, severe cerebral dysfunction is prevalent and can severely impact quality of life [3]. Data on long-term OHCA prognostication are limited and typically involve evaluating initial cardiac rhythm, defibrillation timing, and resuscitation quality [4,5,6,7,8,9,10]. Despite the common use of resuscitation duration as prognostic indicators for cerebral disability and mortality, its predictive power is limited to short-term events [11,12,13,14,15]. The potential of circulating biomarkers, indicative of fibroinflammatory response, volume overload, and cardiomyocyte injury to predict outcomes in OHCA patients, has been studied; however, these studies have been limited either to short-term outcomes or have been retrospective analyses that do not thoroughly consider both severe brain damage and overall mortality [16,17,18,19,20,21,22].

Our study examines brain natriuretic peptide (BNP), troponin I, and galectin-3 levels, alongside traditional clinical risk indicators to predict long-term all-cause mortality and cerebral disability in resuscitated OHCA patients. By synthesizing circulating biomarker levels and clinical data, we aim to assess the prognostic value of these biomarkers for long-term prognostication, which can facilitate focused treatment strategies and interventions for OHCA survivors.

## 2. Methods

### 2.1. Study Design and Setting

This is a prospective multicenter study which enrolled OHCA patients who were successfully resuscitated and admitted for further clinical management to one of the four tertiary care hospitals in the Buffalo–Niagara metropolitan area (Gates Vascular Institute, Buffalo General Medical Center, Sisters of Charity, and South Buffalo Mercy Hospital) from 12/2016 to 12/2018. All clinical procedures and protocols conformed to institutional guidelines and were approved by the Institutional review board (IRB) at the University at Buffalo (STUDY00000284. Approval date 9 May 2024) The human studies conformed to the local good clinical research practice guidelines for the protection of human subjects. Clinical data were secured in accordance with HIPAA guidelines to ensure patient confidentiality. 

### 2.2. Patient Enrollment

OHCA patients that were resuscitated using conventional CPR and admitted to one of our participating facilities were screened for inclusion in this study. OHCA was defined as non-traumatic, sudden cardiac arrest occurring within 1 h of the onset of symptoms in an apparently healthy subject. Patients with documented trauma preceding OHCA, acute myocardial infraction (AMI) or cardiac surgery within the preceding three months, or any active malignancy, were excluded. Patients admitted to a participating facility with the diagnosis of ST-elevation myocardial infarction (STEMI) without cardiac arrest were enrolled as acute myocardial infarction controls (AMI control group). Patients with no cardiovascular disease history were recruited for the healthy control group to establish baseline galectin-3 levels in healthy individuals. 

### 2.3. Clinical Data Abstraction

Baseline demographics, clinical characteristics, laboratory values, and electrocardiographic data were collected via a chart review of the OHCA index hospitalization records. Additionally, the events surrounding OHCA including initial ECG rhythm, corrected QT interval, and time-to-resuscitation were retrieved from the emergency medical services dataset.

### 2.4. Biomarkers Measurement

Serum biochemical data including initial and peak troponin-I levels, creatinine kinase (CK), arterial pH, and brain natriuretic peptide (BNP) were collected at presentation as part of standard care. Galectin-3 sample collection and measurement was performed using our previously published standardized protocol [23].

### 2.5. Outcome Measures

Patients were followed for the primary outcome of all-cause mortality from their index hospitalization until 31 December 2023. Severe cerebral disability (defined as a cerebral performance category score of equal to or greater than 3) was assessed, which is a well-validated prognostic algorithm commonly used in clinical practice [24]. Cerebral performance category scores of 3–5 were defined as poor neurological outcomes [25]. Outcome events were obtained from medical chart review, follow-up with participants’ primary care provider, or by standardized telephone interviews with the patient or their family members.

### 2.6. Statistical Analysis 

Continuous data were expressed either as mean ± standard deviation or median with interquartile range (IQR). Categorical data were expressed as percentage (%). A Chi-square (χ^2^) test or Fisher exact test was used to compare categorical variables. Student’s t-tests were used for continuous data with normal distribution. Correlation between variables was assessed using Spearman’s correlation.

Univariate Cox proportional hazard regression models were constructed to establish the association between the primary outcome of long-term all-cause mortality and clinical variables. Log-transformed values for galectin-3, troponin-I, and BNP were used to ensure normality. Significant variables from univariate regression were used to define risk factors used for patient stratification in subsequent survival analyses. Significant variables from univariate analysis were entered into a multivariate Cox regression model to identify independent predictors of long-term mortality. 

Simple logistic regression was used to examine the association between variables and neurological outcome. Nested multiple linear regression models were constructed and compared using the extra sum-of-squares F test for predicting long-term cerebral disability.

Receiver operating characteristic (ROC) curves were utilized to assess the prognostic value of variables in predicting outcome, and optimal cut points for variables with a significant area-under-the-curve (AUC) were derived using the maximized Youden index. 

Kaplan–Meier (KM) survival curves were constructed based on risk factors and prognostic thresholds of significant biomarkers and compared using log-rank statistics. Cox proportional hazard regression models were constructed and compared to a reference model, which included age and sex, to assess the incremental prognostic value of significant variables. We used Akaike information criterion (AIC) to compare model fits and assessed discrimination using Harrell’s concordance statistic (c-statistic). Data analysis was performed using GraphPad Prism 10.1.1 (GraphPad Software, Inc., San Diego, CA, USA).

## 3. Results

### 3.1. Patient Population

Between 1 December 2016 and 31 December 2018, 144 OHCA patients, 30 AMI controls, and 30 healthy controls were enrolled. The mean age of the OHCA cohort was 60.8 ± 14.4 years; 34.7% were females and 41.3% had asystole as the initial rhythm. The mean time from cardiac arrest to sample collection for biomarker analysis was 255 ± 363.2 min. Galectin-3 was significantly higher in the OHCA cohort compared to the AMI control group and healthy controls (OHCA: 31.48 ng/mL (IQR: 17.25–52.60), AMI: 12.68 ng/mL (IQR 9.61–17.39), Control: 2.09 ng/mL (IQR: 1.56–2.64), *p* < 0.0001). Serum BNP was higher in the OHCA group compared to AMI (695.6 ± 1213.8 vs. 259.6 ± 246.1). Conversely, peak troponin was higher in the AMI group. Compared to the AMI group, the OHCA cohort was less likely to have a history of prior MI but was more likely to have CKD. Table 1 compares variables for the OHCA versus AMI cohorts.

### 3.2. Biomarkers and Mortality

**Figure 1:**
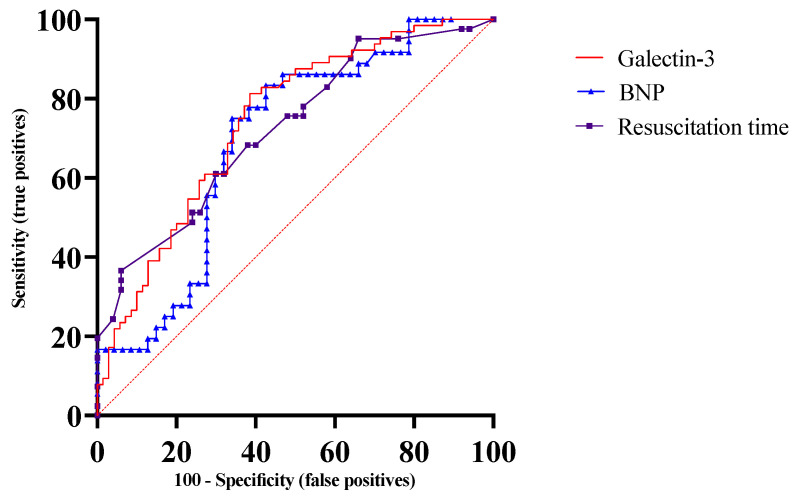
**Combined receiver operating characteristics (ROC) curves for predicting in-hospital mortality.** ROC analysis showed an area-under-the-curve (AUC) for galectin-3 of 0.74 (*p* < 0.0001), AUC for resuscitation time of 0.72 (*p* = 0.0003), AUC for BNP 0.69 (*p* = 0.002), AUC for QTc of 0.65 (*p* = 0.002), and AUC for pH of 0.63 (*p* = 0.012). BNP = brain natriuretic peptide.

Among successfully resuscitated OHCA patients, 68 (47.2%) died during index hospitalization and 76 (52.8%) survived to discharge. The in-hospital mortality group was more likely to have a history of prior diuretic use (*p* = 0.02). There was no significant difference in initial and peak troponin-I, CK, and lactate levels between patients with in-hospital mortality versus survivors. Patients who died had a significantly higher galectin-3, higher BNP, lower pH, longer resuscitation time, and longer QTc compared to survivors (41.3 ng/mL (IQR 28.23–68.48) vs. 20.95 ng/mL (IQR 9.8–38.68), *p* < 0.0001; 1044 ± 1576 pg/mL vs. 483.4 ± 776.1 pg/mL, *p* = 0.04; 7.13 ± 0.17 vs. 7.21 ± 0.15, *p* = 0.01; 21.2 ± 30.4 min vs. 9.5 ± 8.1 min, *p* = 0.0005; 456.6 ± 50.3 ms vs. 434.3 ± 56.3 ms, *p* = 0.01, respectively). ROC analysis for predicting in-hospital deaths showed that galectin-3 had the highest AUC (0.74, *p* < 0.0001) for predicting mortality (Figure 1).

During the median follow-up of 5.7 years (IQR 2.9–6.6), 87 resuscitated patients died. The highest mortality was noted within the first 4 weeks (47%), with a 25% mortality after discharge. Table 2 compares variables for survivors versus non-survivors. Univariate Cox-proportional hazard analysis identified elevated galectin-3, elevated BNP, and prolonged resuscitation time as significant risk factors associated with increased long-term mortality (Table 3). Initial and peak troponin-I were not significantly associated with long-term mortality. For multivariate analysis, 52 patients were excluded due to missing data. Analysis performed on 92 subjects (survivors = 43; non-survivors = 49) showed that galectin-3 remained an independent predictor of long-term mortality in models adjusted for significant variables from the univariate analysis, including BNP, arterial pH, and resuscitation time. Table 4 summarizes results of the multivariate analyses. ROC analysis for long-term death showed significant AUC for galectin-3 (0.74, *p* < 0.0001), BNP (0.79, *p* < 0.0001), and resuscitation time (0.72, *p* = 0.0003). Based on maximized Youden index, galectin-3 > 26.3 ng/mL (sensitivity 75%, specificity 68%), BNP > 140 pg/mL, and resuscitation time > 11.5 min were associated with significantly increased mortality.

### 3.3. Biomarkers and Severe Cerebral Disability

Simple logistic regression showed that galectin-3, BNP, arterial pH, and resuscitation time were significantly associated with long-term poor cerebral outcome (log likelihood ratio: 10.05, *p* = 0.001; 7.88, *p* = 0.005; 4.82, *p* = 0.028; 10.3, *p* = 0.001 respectively), whereas troponin-I was not. In multiple linear regression, a model containing galectin-3 outperformed arterial pH and resuscitation time in predicting poor cerebral outcome (AIC: 72.2 vs. 76.2 and 58.4 vs. 66.4, respectively). A prediction model with BNP also outperformed arterial pH (AIC: 53.37 vs. 54.95); however, it did not perform better than resuscitation time for predicting severe cerebral disability (AIC: 47.9 vs. 44.5). In comparing galectin-3 and BNP, a model with galectin-3 had a better fit compared to BNP for predicting severe cerebral disability (AIC: 56.9 vs. 59.9). 

### 3.4. Incremental Prognostic Value of Biomarkers

**Figure 2:**
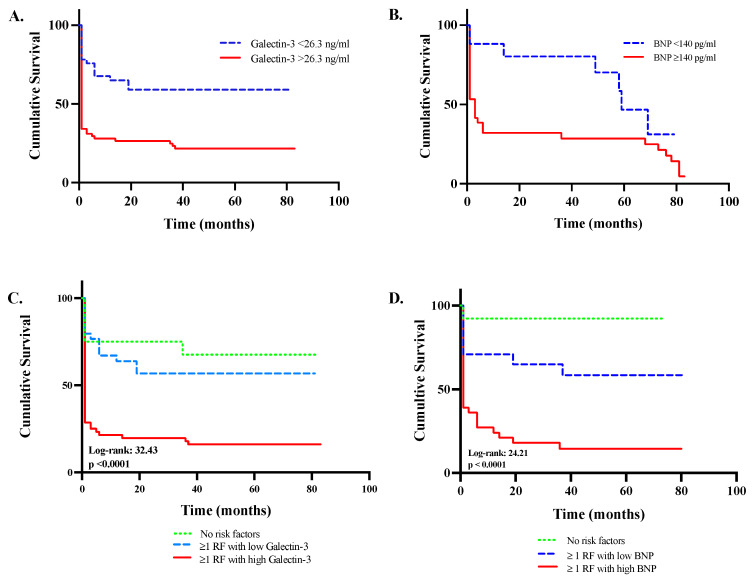
**Combinatorial analysis of all-cause mortality using Kaplan–Meier survival plot in resuscitated out-of-hospital cardiac arrest patients (OHCA).** Cumulative survival based on prognostic thresholds of galectin-3 (**A**) and BNP (**B**) levels. Survival plots compared based on presence of risk factors in combination with serum levels of galectin-3 (**C**) and BNP (**D**) above and below their respective prognostic thresholds. Risk factors are significant variables derived from univariate analysis including the following: (1) use of diuretics or statins prior to OHCA, (2) history of diabetes or CKD, (3) age > 66.5 years, (4) resuscitation time of >11.5 min, (5) initial rhythm asystole, (6) hemoglobin level < 13.5 mg/dL, (7) eGFR < 42 mL/min/1.73 m^2^, (8) arterial pH < 7.1, or (9) QTc > 440 ms. BNP = brain natriuretic peptide; CKD = chronic kidney disease; OHCA = out-of-hospital cardiac arrest; RF = risk factor.

Kaplan–Meier survival analysis showed that subjects with galectin-3 > 26.3 ng/mL had significantly higher long-term mortality compared to those with lower galectin-3, independent of other risk factors (HR 2.49 (1.6–3.9), *p* < 0.0001). When subjects were stratified based on other risk factor only, there was no difference in rates of death between subjects with and without risk factors (HR 1.24 (0.5–3.1), *p* = 0.6). When elevated galectin-3 was combined with other risk factors, the death rate was significantly elevated (HR 3.15 (1.78–5.58), *p* < 0.0001). Among subjects with risk factors, the presence of elevated galectin-3 (>26.3 ng/mL) was still significantly associated with higher mortality rates (HR 2.71 (1.7–4.3), *p* < 0.0001). Similarly, subjects with BNP > 140 pg/mL had an increased risk of death (HR 2.63 (1.44–4.80), *p* = 0.002). When elevated BNP was combined with other risk factors, the risk of death was also elevated (HR 2.66 (1.44–4.92), *p* = 0.0004) (Figure 2). Cox proportional hazard regression models showed that compared to the reference model, incorporating BNP, resuscitation time, galectin-3, or arterial pH resulted in an improved model fit. The incorporation of galectin-3 demonstrated the largest improvement in model diagnostics compared to other variables (Table 5). 

Comparative nested multiple linear regression was used to assess the incremental value of galectin-3 and BNP in predicting cerebral disability. Analysis showed that a model containing galectin-3 in addition to resuscitation time predicted severe cerebral disability better than a model with resuscitation time alone (F = 11.37, *p* = 0.001). Similarly, galectin-3 plus arterial pH performed better than arterial pH alone (F = 5.34, *p* = 0.023). BNP combined with either resuscitation time or arterial pH also performed better than either alone (F = 5.37, *p* = 0.026; F = 4.23, *p* = 0.044, respectively). When galectin-3 was combined with BNP, the resulting model performed significantly better than BNP alone, but not than galectin-3 alone (F = 5.96, *p* = 0.018; F = 2.95, *p* = 0.092, respectively). 

## 4. Discussion

Here, we report a prospective analysis of biochemical and clinical markers for predicting long-term mortality and cerebral disability in a post-cardiac arrest community cohort. Notably, galectin-3 was able to identify patients at the highest risk of in-hospital death better than other established prognostic factors. Galectin-3 was also significantly associated with long-term mortality and, importantly, the association remained strong in models adjusted for key clinical variables, suggesting an independent association between galectin-3 and long-term mortality. Conversely, while other traditional markers such as arterial pH, BNP, and resuscitation time were associated with death in the univariate model, they did not retain significance in the adjusted model. Published literature regarding the prognostic utility of these biomarkers and clinical variables is inconsistent [9,13,14,15,17], which is likely a reflection of the heterogeneity and complex interplay of the multiple variables that define post-arrest syndrome. Our results, along with prior reports, suggest there may be a role for these biomarkers in a multimodal approach for post-arrest prognostication. We did not observe a significant association between troponin-I and the risk of mortality or cerebral dysfunction. While this might seem counterintuitive, it agrees with several published studies [20,26,27]. A possible explanation for this can be that myocardial injury is not a direct determinant of death following cardiac arrest [28,29,30]. Additionally, other factors besides acute coronary occlusion such as resuscitation time, number of defibrillation shocks, and impaired renal function can lead to troponin-I elevation and thus attenuate its prognostic utility [31,32,33]. 

Survival analyses showed that elevated galectin-3 and BNP were associated with a significantly increased death rate, not only in patients without additional risk factors, but also in patients with established risks for poor outcomes including prolonged resuscitation time and unfavorable serum biochemical profiles. This demonstrates an additive prognostic value of these biomarkers in predicting mortality, beyond traditional risk factors. Furthermore, comparative Cox proportional hazard analysis demonstrated an Incremental value of galectin-3 and BNP in comparison to the reference model. However, only galectin-3 demonstrated a significant improvement in predicting long-term death when added to other risk factors, as evident by an improved model fit and discrimination. It is important to note, however, that while our data showed an incremental value of galectin-3 when combined with other risk factors, the presence of elevated galectin-3 alone was still a good predictor of mortality. Thus, our results suggest both an incremental and an independent role of galectin-3 in prognosticating post-arrest patients. 

Galectin-3, a beta-galactoside-binding lectin produced by activated macrophages and fibroblasts, is reported to be involved in cardiac pathology, affecting inflammation, fibrosis, and cardiomyocyte apoptosis [34]. Its reported link to heart failure, myocardial infarction, and ventricular arrhythmias, wherein elevated levels of galectin-3 correlate with adverse cardiac remodeling and reduced ejection fraction [35,36,37,38,39,40], makes it a potential biomarker for cardiac risk stratification. Additionally, galectin-3 is implicated in several neuroinflammatory and neurodegenerative processes including multiple sclerosis, Alzheimer’s disease, and stroke, highlighting its potential role as a biomarker for neuroprognostication [41,42,43].

Since our first preclinical study published in 2004, identifying increased galectin-3 expression in cardiac fibrosis [35], and the first clinical study published in 2006 showing increased circulating galectin-3 in heart failure [39], several other groups have reported an association between galectin-3 expression and cardiac remodeling, heart failure, and death. However, there is a lack of data examining the association between circulating galectin-3 and post-resuscitation outcomes. The prediction of prognosis following resuscitation remains clinically challenging due to the multitude of variables that can affect the outcome. To date, there is no single test that can predict post-resuscitation mortality with reasonable accuracy, and it is unlikely that one will ever exist given the complex interplay of pathologic processes that define post-arrest syndromes. Thus, an approach incorporating multiple biomarkers to assess multiorgan functionality is crucial. Huang et al. reported a multimarker approach to prognosticate resuscitated patients and found that biomarkers with an inflammatory or anti-inflammatory roles were significantly higher in non-survivors [18]. Our results support this approach and underscore the potential role of incorporating galectin-3 in post-arrest risk stratification algorithms.

Galectin-3 has emerged as a significant player in cardiac pathophysiology, with an increasing body of evidence alluding to its role in arrhythmogenesis. As an established fibroinflammatory mediator, galectin-3 promotes cardiac fibrosis through multiple mechanisms [34], thus providing a substrate for the development and maintenance of potentially fatal arrhythmias [40,44]. Additionally, galectin-3 may be implicated in direct modulation of surface ion channels, potentially influencing electrical conduction and arrhythmia susceptibility [45,46]. While these interactions were reported in the kidneys, a similar mechanism is possible in cardiac tissue [47]. By linking elevated circulating galectin-3 level with increased mortality in cardiac arrest patients, our results reinforce the pathologic mechanisms of galectin-3 and support its putative role in arrhythmogenesis and sudden cardiac death.

Cerebroprognostication after cardiac arrest is performed using a multimodal approach comprising neurological exams, brain imaging, and serum biomarkers. Neuronal damage biomarkers such as neuron-specific enolase (NSE) and neurofilament light (NfL) have been implicated in predicting post-arrest neurological outcomes [48,49]. Additionally, our group recently reported perforin-2 as predictor of short-term poor cerebral disability following cardiac arrest [50]. Other studies have also reported prolonged resuscitation time and elevated BNP as markers of poor neurological outcome [17,51]. In the current study, we report elevated galectin-3 as a predictor of long-term cerebral disability, which is plausible since galectin-3 is highly expressed and released by damaged neuronal cells and is involved in several neurodegenerative conditions [41]. Importantly, our results demonstrated an incremental prognostic value of galectin-3 when combined with other predictors of poor outcomes. Thus, the integration of galectin-3 in cerebroprognostication pathways holds promising clinical significance for post-arrest risk stratification.

Overall, our study highlights the significance of galectin-3 as a strong predictor of long-term mortality and cerebral disability in post-cardiac arrest patients. Galectin-3 showed robust associations with both in-hospital death and long-term outcomes, even when adjusted for other clinical variables. The findings suggest that incorporating galectin-3 into multimodal risk stratification approaches could enhance prognostication accuracy in post-arrest care, particularly given its roles in cardiac pathology and neuroprognostication.

## 5. Limitations

Our study has several limitations. First, the number of participants is modest, and a larger number of patients would strengthen our statistical inferences. Second, missing data were a limitation in our multivariate analyses; however, the data were missing at random and we used complete case analysis to avoid bias. Third, due to the nature of this observational study, we cannot exclude residual confounding effects, although the effect on our results is likely minimal as our models were adjusted for several covariates known to affect the risk of poor outcomes. As in many prospective studies, some patients were lost to follow-up; therefore, we do not have all the survival data at the end of the study period. However, these patients were censored in our statistical analyses and this loss of data did not seem to affect our assessment of the utility of galectin-3 in risk stratification. Lastly, our analysis of biomarkers is not comprehensive since there are other established prognostic markers, such as NSE and NfL, that were not assessed in this study. Regarding strengths, this is the first study to examine galectin-3 as a prognostic biomarker for long-term post-arrest mortality and cerebral dysfunction simultaneously, thus introducing a novel application for this molecule and highlighting its potential role in a multimarker approach for risk stratifying cardiac arrest survivors. Furthermore, because blood samples for biomarker analysis were obtained shortly following cardiac arrest, our results reflect early prognostication, which can guide initial clinical management.

## 6. Conclusions

In conclusion, our results highlight the incremental prognostic role of galectin-3 in predicting long-term outcome in OHCA survivors and support its use as part of a multimarker approach with other traditional biomarkers. Further studies are needed to confirm our results as well as to examine and compare additional biomarkers in predicting post-arrest outcomes.

## Figures and Tables

**Table 1:** Baseline demographic and clinical characteristics by patient cohort.

	AMI (*n* = 30)	OHCA (*n* = 144)	*p* Value
**Baseline Characteristics**			
Age, years	64.4 ± 11.7	60.8 ± 14.4	0.28
Gender, % females	40	34.7	0.67
**Risk Factors**			
HTN, %	77.8	59.3	0.19
DM, %	22.2	30.0	0.59
CKD, %	0	18.6	***0.04***
Smoking, %	50	51.8	1.0
Atrial fibrillation, %	22.2	11.4	0.25
Family history of cardiac arrest, %	11.1	0.72	***0.03***
Prior AMI, %	38.9	16.4	***0.04***
Prior coronary revascularization, %	44.4	17.2	***0.012***
**Medications**			
Aspirin, %	20.0	30.0	0.3
Statin, %	55.6	42.2	0.31
Beta Blocker, %	61.1	39.3	0.13
Aldosterone blocker, %	0.0	3.6	1.00
ACE/ARBi, %	50.0	32.9	0.18
Diuretics, %	16.7	25.9	0.56
**Laboratory Values**			
Hemoglobin, g/dL	13.3 ± 1.5	12.9 ± 2.3	0.47
Creatinine, mg/dL	1.0 ± 0.3	1.9 ± 1.9	***0.0005***
eGFR. mL/min/1.73 m^2^	56.4 ± 5.7	48.0 ± 20.4	***0.02***
BNP, ng/mL	259.6 ± 246.1	695.6 ± 1213.8	0.47
Initial CK, U/L	448.0 ± 538.6	1121.5 ± 1649.2	0.10
CK, U/L	76.6 ± 151.9	95.1 ± 218.3	0.90
Peak troponin, ng/mL	79.3 ± 104.2	28.3 ± 114.4	***0.02***
Serum galectin-3, ng/mL, median (IQR)	12.68 (9.61–17.39)	31.48 (17.25–52.60)	***<0.0001***
**Echo and ECG Parameters**			
LVEF post-arrest, %	50.2 ± 12.3	48.1 ± 14.1	0.62
LVEF < 35%, %	11.8	24.1	0.36
QTc on initial ECG, ms	409.4 ± 40.6	446.0 ± 53.6	***0.005***
**Outcomes**			
All-cause mortality, %	10.0	60.4	***<0.0001***

Values are mean ± SD or median (IQR: interquartile range). HTN = hypertension; DM = diabetes mellitus; CKD = chronic kidney disease; AMI = acute myocardial infarction; OHCA = out-of-hospital cardiac arrest; ACEI = angiotensin-converting enzyme inhibitor; ARB = angiotensin II receptor blocker; eGFR = estimated glomerular filtration rate; ECG = electrocardiogram; BNP = brain natriuretic peptide; IQR = interquartile range; PEA = pulseless electrical activity; CK = creatinine kinase; LVEF = left ventricular ejection fraction; QTc = corrected QT interval. Bolded italicized *p*-values denote statistical significance.

**Table 2:** Patient characteristics by long-term survival following resuscitated out-of-hospital cardiac arrest.

	Survivors (*n* = 57)	Non-Survivors (*n* = 87)	*p*-Value
**Baseline Characteristics**			
Age, years	56.5 ± 13.5	63.6 ± 14.3	***0.003***
Female gender, %	31.5	36.7	0.59
**Medications on Presentation**			
Aspirin, %	32.4	26.1	0.46
Statin, %	32.4	52.2	***0.03***
Beta Blocker, %	38.0	40.6	0.86
ACEI/ARB, %	33.8	31.9	0.86
Diuretic, %	11.4	40.6	***<0.0001***
Aldosterone blocker, %	3.6	5.8	0.21
**Risk Factors**			
HTN, %	59.2	59.4	1.00
DM, %	21.1	39.1	***0.02***
Smoking, %	47.9	55.9	0.39
Prior AMI, %	12.7	20.3	0.26
CKD, %	9.9	27.5	***0.008***
Atrial fibrillation, %	9.9	13.0	0.60
Prior coronary revascularization, %	16.9	17.4	1.00
Family history of SCA, %	1.4	0	1.00
**Laboratory Values**			
Hemoglobin, g/dL	13.7 ± 1.8	12.0 ± 2.5	***<0.0001***
Creatinine, mg/dL	1.5 ± 1.1	2.2 ± 2.5	***0.04***
eGFR, mL/min/1.73 m^2^	54.16 ± 12.93	44.1 ± 22.2	***0.008***
BNP, pg/mL	231 ± 347	1090 ± 1481	***<0.0001***
CK, U/L	1160.6 ± 1539.3	1080.1 ± 1773.5	0.64
Peak troponin, ng/mL	19.7 ± 34.8	33.9 ± 143.7	0.17
Serum galectin-3, ng/mL, median (IQR)	20.3 (9.2–34.7)	40.1 (26.6–58.7)	***<0.0001***
Time from event to galectin 3 sample, mins	295 ± 412.9	213.1 ± 297.1	0.56
**Echo and EKG Parameters**			
LVEF post-arrest, %	48. 2 ± 12.6	48.1 ± 15.5	0.57
LVEF, <35%, %	21.4	26.9	0.54
QTc on initial EKG, ms	432.8 ± 51.9	460.1 ± 52.1	***0.001***
**Event Variables**			
Duration of resuscitation, mins	8.8 ± 8.6	19.2 ± 18.6	***0.0001***
Initial Asystole/PEA rhythm, %	19.7	64.2	***0.001***

Values are mean ± SD or median (IQR: interquartile range). HTN = hypertension; AMI = acute myocardial infarction; ACEI = angiotensin-converting enzyme inhibitor; ARB = angiotensin II receptor blocker; IQR = interquartile range; SCA = sudden cardiac death; PEA = pulseless electrical activity; LVEF = left ventricular ejection fraction. Bolded italicized *p*-values denote statistical significance.

**Table 3:** Significant risk factors from univariate analysis associated with all-cause mortality following resuscitated out-of-hospital cardiac arrest.

Variable	Hazard Ratio (95% CI)	*p*-Value
Age	1.02 (1.004–1.037)	0.010
Galectin-3	5.79 (2.88–12.04)	<0.0001
BNP	2.53 (1.67–3.93)	<0.0001
Hemoglobin	0.83 (0.75–0.91)	<0.0001
eGFR	0.987 (0.97–0.99)	0.030
Arterial pH	0.19 (0.05–0.77)	0.010
Initial rhythm asystole	4.21 (2.59–7.06)	<0.0001
Resuscitation time	1.022 (1.009–1.032)	<0.0001
QTc	1.004 (1.001–1.007)	0.009
History of diabetes	1.83 (1.17–2.82)	0.006
History of CKD	1.716 (1.032–2.74)	0.029
History of diuretic use	2.42 (1.55–3.75)	<0.0001
History of statin use	1.74 (1.13–2.68)	0.010

BNP = brain natriuretic peptide; CKD = chronic kidney disease; 95% CI = 95% confidence interval.

**Table 4:** Adjusted multivariate analysis for the primary outcome of long-term mortality following resuscitated out-of-hospital cardiac arrest. Analysis performed on 92 subjects (survivors = 43; non-survivors = 49).

Predictor	Hazard Ratio	95% CI	*p*-Value
**Galectin-3**	15.93	1.49–191.4	***p* = 0.022**
**Resuscitation time**	1.02	0.97–1.08	*p* = 0.372
**BNP**	1.17	0.43–3.27	*p* = 0.745
**Arterial pH**	0.37	0.001–17.58	*p* = 0.629

95% CI = 95% confidence interval.

**Table 5:** Comparative prognostic value of biomarkers and clinical variables in predicting long-term mortality following resuscitated out-of-hospital cardiac arrest.

	Reference Model	Reference + Risk Factor(s)	Reference Model + BNP	Reference Model + pH	Reference Model + Resuscitation Time	Reference Model + Galectin-3	[Reference Model + Risk Factor(s)] + BNP	[Reference Model + Risk Factor(s)] + Galectin-3
**Log-likelihood ratio**	-	*p* = 0.009	*p* = 0.01	*p* = 0.014	*p* = 0.003	*p* < 0.0001	*p* = 0.005	*p* < 0.0001
AIC	701.3	697.8	698.6	698.7	695.3	679.8	696.4	676.4
Partial LL	−348.7	−345.9	−346.3	−346.3	−343.6	−336.9	−344.2	−334.2
**Discrimination**								
C-statistic	0.59 (0.50–0.67)	0.61 (0.53–0.70)	0.62 (0.54–0.71)	0.62 (0.54–0.71)	0.65 (0.56–0.73)	0.73 (0.65–0.80)	0.63 (0.55–0.72)	0.74 (0.67–0.82)

BNP = brain natriuretic peptide; AIC = Akaike information criterion; LL = log likelihood.

## Data Availability

The original contributions presented in the study are included in the article, further inquiries can be directed to the corresponding authors.

## References

[1] Mozaffarian D., Benjamin E.J., Go A.S., Arnett D.K., Blaha M.J., Cushman M., Das S.R., de Ferranti S., Després J.P., Fullerton H.J. (2016). Heart Disease and Stroke Statistics-2016 Update: A Report From the American Heart Association. Circulation.

[2] Garcia R.A., Girotra S., Jones P.G., McNally B., Spertus J.A., Chan P.S., CARES Surveillance Group (2022). Variation in Out-of-Hospital Cardiac Arrest Survival Across Emergency Medical Service Agencies. Circ. Cardiovasc. Qual. Outcomes.

[3] Geocadin R.G., Agarwal S., Goss A.L., Callaway C.W., Richie M. (2023). Cardiac Arrest and Neurologic Recovery: Insights from the Case of Mr. Damar Hamlin. Ann. Neurol..

[4] Fugate J.E., Brinjikji W., Mandrekar J.N., Cloft H.J., White R.D., Wijdicks E.F., Rabinstein A.A. (2012). Post-cardiac arrest mortality is declining: A study of the US National Inpatient Sample 2001 to 2009. Circulation.

[5] Buick J.E., Drennan I.R., Scales D.C., Brooks S.C., Byers A., Cheskes S., Dainty K.N., Feldman M., Verbeek P.R., Zhan C. (2018). Improving Temporal Trends in Survival and Neurological Outcomes After Out-of-Hospital Cardiac Arrest. Circ. Cardiovasc. Qual. Outcomes.

[6] Chan P.S., McNally B., Tang F., Kellermann A., CARES Surveillance Group (2014). Recent trends in survival from out-of-hospital cardiac arrest in the United States. Circulation.

[7] Lee D.H., Cho I.S., Lee S.H., Min Y.I., Min J.H., Kim S.H., Lee Y.H., Korean Hypothermia Network Investigators (2015). Correlation between initial serum levels of lactate after return of spontaneous circulation and survival and neurological outcomes in patients who undergo therapeutic hypothermia after cardiac arrest. Resuscitation.

[8] Sodeck G.H., Domanovits H., Sterz F., Schillinger M., Losert H., Havel C., Kliegel A., Vlcek M., Frossard M., Laggner A.N. (2007). Can brain natriuretic peptide predict outcome after cardiac arrest? An observational study. Resuscitation.

[9] von Auenmueller K.I., Christ M., Sasko B.M., Trappe H.J. (2017). The Value of Arterial Blood Gas Parameters for Prediction of Mortality in Survivors of Out-of-hospital Cardiac Arrest. J. Emerg. Trauma Shock.

[10] Wibrandt I., Norsted K., Schmidt H., Schierbeck J. (2015). Predictors for outcome among cardiac arrest patients: The importance of initial cardiac arrest rhythm versus time to return of spontaneous circulation, a retrospective cohort study. BMC Emerg. Med..

[11] Mørk S.R., Bøtker M.T., Christensen S., Tang M., Terkelsen C.J. (2022). Survival and neurological outcome after out-of-hospital cardiac arrest treated with and without mechanical circulatory support. Resusc. Plus.

[12] Reynolds J.C., Frisch A., Rittenberger J.C., Callaway C.W. (2013). Duration of resuscitation efforts and functional outcome after out-of-hospital cardiac arrest: When should we change to novel therapies?. Circulation.

[13] Chai J., Fordyce C.B., Guan M., Humphries K., Hutton J., Christenson J., Grunau B. (2023). The association of duration of resuscitation and long-term survival and functional outcomes after out-of-hospital cardiac arrest. Resuscitation.

[14] Kiehl E.L., Amuthan R., Adams M.P., Love T.E., Enfield K.B., Gimple L.W., Cantillon D.J., Menon V. (2019). Initial arterial pH as a predictor of neurologic outcome after out-of-hospital cardiac arrest: A propensity-adjusted analysis. Resuscitation.

[15] Mueller M., Grafeneder J., Schoergenhofer C., Schwameis M., Schriefl C., Poppe M., Clodi C., Koch M., Sterz F., Holzer M. (2021). Initial Blood pH, Lactate and Base Deficit Add No Value to Peri-Arrest Factors in Prognostication of Neurological Outcome After Out-of-Hospital Cardiac Arrest. Front. Med..

[16] Mosleh W., Kattel S., Bhatt H., Al-Jebaje Z., Khan S., Shah T., Dahal S., Khalil C., Frodey K., Elibol J. (2019). Galectin-3 as a Risk Predictor of Mortality in Survivors of Out-of-Hospital Cardiac Arrest. Circ. Arrhythmia Electrophysiol..

[17] Dutta A., Alirhayim Z., Masmoudi Y., Azizian J., McDonald L., Jogu H.R., Qureshi W.T., Majeed N. (2022). Brain Natriuretic Peptide as a Marker of Adverse Neurological Outcomes Among Survivors of Cardiac Arrest. J. Intensive Care Med..

[18] Huang C.-H., Tsai M.-S., Chien K.-L., Chang W.-T., Wang T.-D., Chen S.-C., Ma M.H.-M., Hsu H.-Y., Chen W.-J. (2016). Predicting the outcomes for out-of-hospital cardiac arrest patients using multiple biomarkers and suspension microarray assays. Sci. Rep..

[19] Spoormans E.M., Lemkes J.S., Janssens G.N., van der Hoeven N.W., Jewbali L.S.D., Dubois E.A., Meuwissen M., Rijpstra T.A., Bosker H.A., Blans M.J. (2024). The Prognostic Value of Troponin-T in Out-of-Hospital Cardiac Arrest Without ST-Segment Elevation: A COACT Substudy. J. Soc. Cardiovasc. Angiogr. Interv..

[20] Røsjø H., Vaahersalo J., Hagve T.A., Pettilä V., Kurola J., Omland T., FINNRESUSCI Laboratory Study Group (2014). Prognostic value of high-sensitivity troponin T levels in patients with ventricular arrhythmias and out-of-hospital cardiac arrest: Data from the prospective FINNRESUSCI study. Crit. Care.

[21] Sasson C., Rogers M.A., Dahl J., Kellermann A.L. (2010). Predictors of survival from out-of-hospital cardiac arrest: A systematic review and meta-analysis. Circ. Cardiovasc. Qual. Outcomes.

[22] Berdowski J., Berg R.A., Tijssen J.G., Koster R.W. (2010). Global incidences of out-of-hospital cardiac arrest and survival rates: Systematic review of 67 prospective studies. Resuscitation.

[23] Mosleh W., Chaudhari M.R., Sonkawade S., Mahajan S., Khalil C., Frodey K., Shah T., Dahal S., Karki R., Katkar R. (2018). The Therapeutic Potential of Blocking Galectin-3 Expression in Acute Myocardial Infarction and Mitigating Inflammation of Infarct Region: A Clinical Outcome-Based Translational Study. Biomark. Insights.

[24] Chan P.S., Berg R.A., Tang Y., Curtis L.H., Spertus J.A., American Heart Association’s Get With the Guidelines–Resuscitation Investigators (2016). Association Between Therapeutic Hypothermia and Survival After In-Hospital Cardiac Arrest. JAMA.

[25] Maupain C., Bougouin W., Lamhaut L., Deye N., Diehl J.L., Geri G., Perier M.C., Beganton F., Marijon E., Jouven X. (2016). The CAHP (Cardiac Arrest Hospital Prognosis) score: A tool for risk stratification after out-of-hospital cardiac arrest. Eur. Heart J..

[26] Agusala V., Khera R., Cheeran D., Mody P., Reddy P.P., Link M.S. (2019). Diagnostic and prognostic utility of cardiac troponin in post-cardiac arrest care. Resuscitation.

[27] Geri G., Mongardon N., Dumas F., Chenevier-Gobeaux C., Varenne O., Jouven X., Vivien B., Mira J.P., Empana J.P., Spaulding C. (2013). Diagnosis performance of high sensitivity troponin assay in out-of-hospital cardiac arrest patients. Int. J. Cardiol..

[28] Laver S., Farrow C., Turner D., Nolan J. (2004). Mode of death after admission to an intensive care unit following cardiac arrest. Intensive Care Med..

[29] Lemiale V., Dumas F., Mongardon N., Giovanetti O., Charpentier J., Chiche J.D., Carli P., Mira J.P., Nolan J., Cariou A. (2013). Intensive care unit mortality after cardiac arrest: The relative contribution of shock and brain injury in a large cohort. Intensive Care Med..

[30] Dragancea I., Rundgren M., Englund E., Friberg H., Cronberg T. (2013). The influence of induced hypothermia and delayed prognostication on the mode of death after cardiac arrest. Resuscitation.

[31] Omland T., de Lemos J.A., Sabatine M.S., Christophi C.A., Rice M.M., Jablonski K.A., Tjora S., Domanski M.J., Gersh B.J., Rouleau J.L. (2009). A sensitive cardiac troponin T assay in stable coronary artery disease. N. Engl. J. Med..

[32] Grubb N.R., Fox K.A.A.A., Cawood P. (1996). Resuscitation from out-of-hospital cardiac arrest: Implication for cardiac enzyme estimation. Resuscitation.

[33] Lin C.C., Chiu T.F., Fang J.Y., Kuan J.T., Chen J.C. (2006). The influence of cardiopulmonary resuscitation without defibrilliation on serum levels of cardiac enzymes: A time course study of out-of-hospital cardiac arrest survivors. Resuscitation.

[34] Frunza O., Russo I., Saxena A., Shinde A.V., Humeres C., Hanif W., Rai V., Su Y., Frangogiannis N.G. (2016). Myocardial Galectin-3 Expression Is Associated with Remodeling of the Pressure-Overloaded Heart and May Delay the Hypertrophic Response without Affecting Survival, Dysfunction, and Cardiac Fibrosis. Am. J. Pathol..

[35] Sharma U.C., Pokharel S., van Brakel T.J., van Berlo J.H., Cleutjens J.P., Schroen B., André S., Crijns H.J., Gabius H.J., Maessen J. (2004). Galectin-3 marks activated macrophages in failure-prone hypertrophied hearts and contributes to cardiac dysfunction. Circulation.

[36] Besler C., Lang D., Urban D., Rommel K.P., von Roeder M., Fengler K., Blazek S., Kandolf R., Klingel K., Thiele H. (2017). Plasma and Cardiac Galectin-3 in Patients With Heart Failure Reflects Both Inflammation and Fibrosis: Implications for Its Use as a Biomarker. Circ. Heart Fail..

[37] Lee K.N., Kim D.Y., Boo K.Y., Kim Y.G., Roh S.Y., Baek Y.S., Kim D.H., Lee D.I., Shim J., Choi J.I. (2022). Therapeutic implications of galectin-3 in patients with atrial fibrillation. Sci. Rep..

[38] Li M., Yuan Y., Guo K., Lao Y., Huang X., Feng L. (2020). Value of Galectin-3 in Acute Myocardial Infarction. Am. J. Cardiovasc. Drugs.

[39] van Kimmenade R.R., Januzzi J.L., Ellinor P.T., Sharma U.C., Bakker J.A., Low A.F., Martinez A., Crijns H.J., MacRae C.A., Menheere P.P. (2006). Utility of amino-terminal pro-brain natriuretic peptide, galectin-3, and apelin for the evaluation of patients with acute heart failure. J. Am. Coll. Cardiol..

[40] Oz F., Onur I., Elitok A., Ademoglu E., Altun I., Bilge A.K., Adalet K. (2017). Galectin-3 correlates with arrhythmogenic right ventricular cardiomyopathy and predicts the risk of ventricular -arrhythmias in patients with implantable defibrillators. Acta Cardiol..

[41] Soares L.C., Al-Dalahmah O., Hillis J., Young C.C., Asbed I., Sakaguchi M., O’Neill E., Szele F.G. (2021). Novel Galectin-3 Roles in Neurogenesis, Inflammation and Neurological Diseases. Cells.

[42] García-Revilla J., Boza-Serrano A., Espinosa-Oliva A.M., Soto M.S., Deierborg T., Ruiz R., de Pablos R.M., Burguillos M.A., Venero J.L. (2022). Galectin-3, a rising star in modulating microglia activation under conditions of neurodegeneration. Cell Death Dis..

[43] Zhuang J.J., Zhou L., Zheng Y.H., Ding Y.S. (2021). The serum galectin-3 levels are associated with the severity and prognosis of ischemic stroke. Aging.

[44] Makimoto H., Müller P., Denise K., Clasen L., Lin T., Angendohr S., Schmidt J., Brinkmeyer C., Kelm M., Bejinariu A. (2022). Clinical Impact of Circulating Galectin-3 on Ventricular Arrhythmias and Heart Failure Hospitalization Independent of Prior Ventricular Arrhythmic Events in Patients with Implantable Cardioverter-defibrillators. Intern. Med..

[45] Pricci F., Leto G., Amadio L., Iacobini C., Romeo G., Cordone S., Gradini R., Barsotti P., Liu F.-T., Di Mario U. (2000). Role of galectin-3 as a receptor for advanced glycosylation end products. Kidney Int..

[46] Huang C.-L. (2010). Regulation of ion channels by secreted Klotho: Mechanisms and implications. Kidney Int..

[47] Takemoto Y., Ramirez R.J., Yokokawa M., Kaur K., Ponce-Balbuena D., Sinno M.C., Willis B.C., Ghanbari H., Ennis S.R., Guerrero-Serna G. (2016). Galectin-3 regulates atrial fibrillation remodeling and predicts catheter ablation outcomes. JACC Basic Transl. Sci..

[48] Humaloja J., Ashton N.J., Skrifvars M.B. (2022). Brain Injury Biomarkers for Predicting Outcome After Cardiac Arrest. Crit. Care.

[49] Hoiland R.L., Rikhraj K.J.K., Thiara S., Fordyce C., Kramer A.H., Skrifvars M.B., Wellington C.L., Griesdale D.E., Fergusson N.A., Sekhon M.S. (2022). Neurologic Prognostication after Cardiac Arrest Using Brain Biomarkers: A Systematic Review and Meta-analysis. JAMA Neurol..

[50] Kattel S., Bhatt H., Xu S., Gurung S., Pokharel S., Sharma U.C. (2020). Macrophage-specific protein perforin-2 is associated with poor neurological recovery and reduced survival after sudden cardiac arrest. Resuscitation.

[51] Goto Y., Funada A., Goto Y. (2016). Relationship Between the Duration of Cardiopulmonary Resuscitation and Favorable Neurological Outcomes After Out-of-Hospital Cardiac Arrest: A Prospective, Nationwide, Population-Based Cohort Study. J. Am. Heart Assoc..

